# The Immunomodulatory and Anti-Inflammatory Role of Polyphenols

**DOI:** 10.3390/nu10111618

**Published:** 2018-11-02

**Authors:** Nour Yahfoufi, Nawal Alsadi, Majed Jambi, Chantal Matar

**Affiliations:** 1 Cellular and Molecular Medicine Department, Faculty of Medicine, University of Ottawa, Ottawa, ON K1H8L1, Canada; nyahf074@uottawa.ca (N.Y.); nalsa068@uottawa.ca (N.A.); mjamb055@uottawa.ca (M.J.); 2 School of Nutrition, Faculty of Health Sciences, University of Ottawa, Ottawa, ON K1H8L1, Canada

**Keywords:** polyphenols, immune system, inflammation, molecular mechanisms, nuclear factor kappa-light-chain-enhancer of activated B cells (NF-κB), arachidonic acid, mitogen-activated protein Kinase (MAPK), cytokines, oxidative stress, reactive oxygen species (ROS), cyclooxygenase (COX), nitric oxide synthase (NOS), lipoxygenase (LOX), superoxide dismutase (SOD), inhibitor of kappa kinase (IKK), extra-cellular signal regulated kinases (ERK), cancer, anti-inflammation, anti-tumorigenic, chronic inflammatory conditions, macrophages, T helper 1 (Th1), Th17, Treg

## Abstract

This review offers a systematic understanding about how polyphenols target multiple inflammatory components and lead to anti-inflammatory mechanisms. It provides a clear understanding of the molecular mechanisms of action of phenolic compounds. Polyphenols regulate immunity by interfering with immune cell regulation, proinflammatory cytokines’ synthesis, and gene expression. They inactivate NF-κB (nuclear factor kappa-light-chain-enhancer of activated B cells) and modulate mitogen-activated protein Kinase (MAPk) and arachidonic acids pathways. Polyphenolic compounds inhibit phosphatidylinositide 3-kinases/protein kinase B (PI3K/AkT), inhibitor of kappa kinase/c-Jun amino-terminal kinases (IKK/JNK), mammalian target of rapamycin complex 1 (mTORC1) which is a protein complex that controls protein synthesis, and JAK/STAT. They can suppress toll-like receptor (TLR) and pro-inflammatory genes’ expression. Their antioxidant activity and ability to inhibit enzymes involved in the production of eicosanoids contribute as well to their anti-inflammation properties. They inhibit certain enzymes involved in reactive oxygen species ROS production like xanthine oxidase and NADPH oxidase (NOX) while they upregulate other endogenous antioxidant enzymes like superoxide dismutase (SOD), catalase, and glutathione (GSH) peroxidase (Px). Furthermore, they inhibit phospholipase A2 (PLA2), cyclooxygenase (COX) and lipoxygenase (LOX) leading to a reduction in the production of prostaglandins (PGs) and leukotrienes (LTs) and inflammation antagonism. The effects of these biologically active compounds on the immune system are associated with extended health benefits for different chronic inflammatory diseases. Studies of plant extracts and compounds show that polyphenols can play a beneficial role in the prevention and the progress of chronic diseases related to inflammation such as diabetes, obesity, neurodegeneration, cancers, and cardiovascular diseases, among other conditions.

## 1. Introduction

Numerous studies have attributed to polyphenols a broad range of biological activities including but not limited to anti-inflammatory, immune-modulatory, antioxidant, cardiovascular protective and anti-cancer actions [1,2,3,4,5]. Polyphenols are ubiquitously made by plants and are present either as glycosides esters or as free aglycones [6]. More than 8000 structural variants exist in the polyphenol family. Polyphenols are bioactive compounds found in fruits and vegetables contributing to their color, flavor, and pharmacological activities [1]. They are classified according to their chemical structures into flavonoids such as flavones, flavonols, isoflavones, neoflavonoids, chalcones, anthocyanidins, and proanthocyanidins and nonflavonoids, such as phenolic acids, stilbenoids, and phenolic amides [7]. The majority of these molecules are metabolites of plants, they are made of several aromatic rings with hydroxyl moieties [8]. Their chemical structures contribute to their classification into different classes. Considering gastrointestinal digestion, some—but not all—polyphenols are absorbed in the small intestine, for example, anthocyanins and the majority of remaining polyphenols except flavonoids are usually stable; these later are unstable in the duodenum. Unabsorbed polyphenols must be hydrolyzed first by digestive enzymes then glycosides with high lipid contents are absorbed by epithelial cells [9,10].

In recent years, consumers prefer using natural food ingredients as additives because of their safety and availability. Applications of phenolic compounds to multiple fresh perishable foods show that they are worthy to be used as preservatives in foods and can be creditable alternatives to synthetic food additives. In this sense, polyphenolic compounds start to substitute chemical additives in food. Different methods like spraying, coating and dipping treatment of food are currently applied in food technology preceding packaging as effective alternatives [11]. Grape seeds and olive oil polyphenols’ rich extracts can be used as food additives for their anti-oxidant properties [12]. Various polyphenols like grape polyphenols demonstrate an efficient role as additives in fish and fish products for their anti-oxidant properties in order to prevent lipid oxidation and quality deterioration of polyunsaturated fatty acids [13]. In addition polyphenolic compounds like flavonols, p-coumaric, and caffeic acids can be used as food preservatives for their antimicrobial activity [11].

Back to inflammation, continuous inflammation is known to be a major cause linked to different human disorders involving cancer, diabetes type II, obesity, arthritis, neurodegenerative diseases, and cardiovascular diseases [14,15]. Polyphenols derived from botanic origin have shown anti-inflammatory activity in vitro and in vivo highlighting their beneficial role as therapeutic tools in multiple acute and chronic disorders [16,17,18,19,20]. Accordingly, many epidemiological and experimental researches have been studying the anti-inflammatory and immune modulation activities of dietary polyphenols [15,21]. The ability of these natural compounds to modify the expression of several pro-inflammatory genes like multiple cytokines, lipoxygenase, nitric oxide synthases cyclooxygenase, in addition to their anti-oxidant characteristics such as ROS (reactive oxygen species) scavenging contributes to the regulation of inflammatory signaling [22,23]. This review will discuss the immunomodulatory effects of dietary polyphenols, their anti-inflammatory abilities, the different mechanisms and pathways involved in reducing inflammation and their contribution to protect from different chronic inflammatory diseases with a focus on their anti-cancer activity.

## 2. Polyphenols and Inflammation

The immune modulation effect of polyphenols is supported by different studies: some polyphenols impact on immune cells populations, modulate cytokines production, and pro-inflammatory genes expression [24,25]. For example, cardioprotective effects of resveratrol present in red wine grape and nuts were mainly attributed to its anti-inflammatory properties. In vivo and in vitro studies demonstrate that resveratrol can inhibit COX, inactivate peroxisome proliferator-activated receptor gamma (PPARγ) and induce eNOS (endothelial nitric oxide synthase) in murine and rat macrophages [26,27,28]. Likewise, a resveratrol analog, RVSA40, inhibits the pro-inflammatory cytokines TNF-α (Tumor necrosis factor alpha) and IL-6 (interleukin-6) in RAW (Murine macrophages cell line) 264.7 macrophages [29]. Another example is the non-flavonoid curcumin found in turmeric plants and mustard. Curcumin was shown to reduce the expression of inflammatory cytokines: TNF and IL-1, adhesion molecules like ICAM-1 (intercellular adhesion molecule-1) and VCAM-1 (vascular cell adhesion molecule-1) in human umbilical vein endothelial cells and inflammatory mediators like prostaglandins and leukotriens. It also inhibits certain enzymes involved in inflammation like COX in mice (cyclooxygenase), LOX (lipoxygenase) in, human endothelial cells MAPK (mitogen-activated protein Kinase), and IKK (inhibitor of kappa kinase). Moreover, curcumin downregulates NF-κB (nuclear factor kappa-light-chain-enhancer of activated B cells) and STAT3 (signal transducer and activator of transcription) and reduces the expression of TLR-2 (toll-like receptor-2) and 4 while, in vivo, it upregulates PPARγ (Peroxisome proliferator-activated receptor gamma) in male adult rats [30,31,32,33,34,35]. Caffeic acid phenethyl ester suppresses TLR4 activation and LPS-mediated NF-κB in macrophages, Quercetin was also shown to inhibit leukotriens biosynthesis in human polymorphonuclear leukocytes [36,37]. COX2 expression is also attenuated by ECGC (Epigallocatechin gallate) in colon cancer cell and androgen-independent PC-3 cells of human prostate cancer, gingerol in and piceatannol (EGCG analog found in Norway spruces) leading to NFκ B inactivation [30,38,39,40]. Furthermore, polyphenols, such as Gingerol and Quercetin can activate the production of adiponectin known for its anti-inflammatory effects [30,39]. Similarily, EGCG blocks NFκ B activation in human epithelial cells and downregulates the expression of iNOS (inducible nitric oxide synthase), NO (nitric oxide) production in macrophages resulting in its immunomodulation [38,40,41]. A series of in vitro studies found that other polyphenols like oleanolic acid, curcumin, kaempferol-3-O-sophoroside, EGCG and lycopene inhibit high mobility group box1 protein, an important chromatin protein that interacts with nucleosomes, transcription factors, and histones regulating transcription and playing a key role in inflammation [35]. All of these examples support the anti-inflammatory effects of polyphenols.

Polyphenols’ use is associated with a direct change in the count and differentiation of specific immune cells. An increase in T helper 1(Th1), natural killer (NK), macrophages and dendritic cells (DCs) in Peyer’s patches and spleen is associated with oral administration of polyphenols extracted from the fruit date in male C3H/HeN mice [24]. In humans, the count of regulatory T cells (Treg or suppressor T cells) characterized by the (CD4 + CD25 + Foxp3+) phenotype and involved in immune tolerance and autoimmune control can be boosted by polyphenols [42,43,44]. In vivo, Epigallocatechin-3-gallate, found in green tea and injected to Laboratory inbred strain (BALB)/c mice, rises the number of functional Treg in spleens, pancreatic lymph nodes, and mesentheric lymph nodes [45]. Similarly, in vitro treatment of Jurkat T cells with EGCG or green tea upsurges the expression of Foxp3 and IL10. Baicalin, a flavone, extracted from Huangqin herb, induces Foxp3 expression in HEK 293 T cells and triggers functional Treg from splenic CD4 + CD25− T cells [46]. Additionally, flavonoids show an agonistic effect of aryl hydrocarbon receptor (AhR) and bind xenobiotic-responsive elements in promoter regions of certain genes, including Foxp3 rising its expression [47].

Th1 and Th17 populations are also affected by polyphenols: EGCG reduces the differentiation of Th1 and reduces the numbers of Th17 and Th9 cells in specific pathogen-free C57/BL6 female mice [48]. Other polyphenols like Baicalin show a reduction of Th17 differentiation in vitro and a diminution of IL-17 expression [49].

Macrophages are affected by polyphenols as well. Macrophages are known to be a key player in the inflammatory response. They initiate inflammation by secreting pro-inflammatory mediators and cytokines like IL-6 and TNF-α [50]. Polyphenols repress macrophages by inhibiting cyclooxygenase-2 (COX-2), inducible nitric oxide synthase (iNOS), thus they reduce the production of TNF-α, interleukine-1-beta (IL-1-β) and IL-6 expression [51]. Chinese propolis [52] containing ferulic acid and coumaric acid, an extract of *Lonicera japónica* Thunb) [53] or *Kalanchoe gracilis* [54] are a good example in this case as per demonstrated by in vitro studies using RAW 264.7 cells.

## 3. Polyphenol and Cytokine Modulation

Cytokines are important mediators’ proteins, essential in networking communication for immune system. Cytokines can be produced by lymphocytes (lymphokines), or monocytes (monokines) with pro-inflammatory and anti-inflammatory effects. Cytokines with chemotactic activities are termed chemokines. The equilibrum between pro-inflammatory cytokines (IL-1β, IL-2, TNFα, Il-6, IL-8, IFN-γ…) and anti-inflammatory cytokines (IL-10, IL-4, TGFβ) are thought to be an important parameter in immune response homeostasis and inflammation underlining many disease [55]. In vivo and in vitro studies demonstrate that polyphenols affect macrophages by inhibiting multiple key regulators of inflammatory response such as the inhibition of TNF-α, IL-1-β, and IL-6 [51].

In humans, consumption of bilberries is associated with a decreased inflammation score in patients’ blood, reflected by decreasing serum levels of IL-6, IL-12, and high sensitivity C reactive protein [56]. Moreover, clinical trials have shown the ability of polyphenol-enriched extra virgin olive oil to reduce IL-6 and C-reactive protein expression in stable coronary heart disease patients [57].

In lipopolysaccharide (LPS)-treated BALB/c mice, a model system of inflammation olive vegetation water show ability to inhibit the production of tumor necrosis factor-alpha usually activated by inflammation [58]. Flavonoids, as well, play an important anti-inflammatory effect by influencing cytokines’ secretion. Several flavonoids are found able to inhibit the expression of various pro-inflammatory cytokines and chemokines like TNFα, IL-1β, IL-6, IL-8, and MCP-1 (monocyte chemoattractant protein-1) in multiple cell types such as LPS-activated mouse primary macrophages, activated human mast cell line, activated human astrocytes, human synovial cells, and human peripheral blood mononuclear cells [59,60,61,62,63,64]. In murine RAW 264.7 macrophages stimulated by LPS, Chinese propolis as well as extract of *Lonicera japónica* Thunb (*Caprifoliaceae*) or *Kalanchoe gracilis* demonstrated inhibitory effects on TNF-α, IL-1-β, and IL-6 [52,53,54]. Similarly, certain polyphenol analogs, like curcumin analog EF31, have shown the ability to inhibit the expression and secretion of TNF-α, IL-1-β, and IL-6 in mouse Raw 264.7 macrophages [65].

Likewise, reduction of the secretion of TNF-α and IL-6 without IL-1β modulation is observed with extracts of chamomile, meadowsweet, willow bark, and isolated polyphenols such as quercetin existing in these extracts in THP1 macrophages [66]. Extract of *Cydonia oblonga* inhibits TNF-α and Interleukin 8 while it increases IL-10 and IL-6 in THP-1monocytes stimulated with LPS. The reduction in TNF-α levels limits the acute inflammatory response [67,68]. Other cytokines like IFNγ might also be inhibited by certain polyphenols. For example, kaempferol reduces the production of IFN-γ in a dose-dependent manner in spleen cells and T cell lines [69].

Certain polyphenols exert their effects on the balance between pro- and anti-inflammatory cytokines production such as quercetin and catechins, they enhance IL-10 release while they inhibit TNFα and IL-1β [59,70]. Extract of *Cydonia oblonga* also inhibits the effects of TNF-α and Interleukin 8 (IL-8) while it raises IL-10 in the same type of monocytes [67,68]. Modulation of inflammatory cytokines is one of many common mechanisms by which polyphenols in general exert their immunomodulatory effects.

## 4. Polyphenols, Inflammation, and Modulation of Different Signaling Pathways

### 4.1. NFκ B Signaling Pathway

NF-κB or nuclear factor kappa-light-chain-enhancer of activated B cells is a complex protein that plays a key role in deoxyribonucleic acid (DNA) transcription, cytokine production and cell survival. It controls immune, inflammation, stress, proliferation and apoptotic responses of a cell to multiple stimuli [58].

The expression of a large number of genes involved in inflammation is controlled by NF-κB such as COX-2, VEGF (vascular endothelial growth Factor), pro-inflammatory cytokines (IL-1, IL-2, IL-6, and TNFα), chemokines (e.g., IL-8, MIP-1α, and MCP-1), adhesion molecules, immuno-receptors, growth factors, and other agents involved in proliferation and invasion [71].

**Figure 1:**
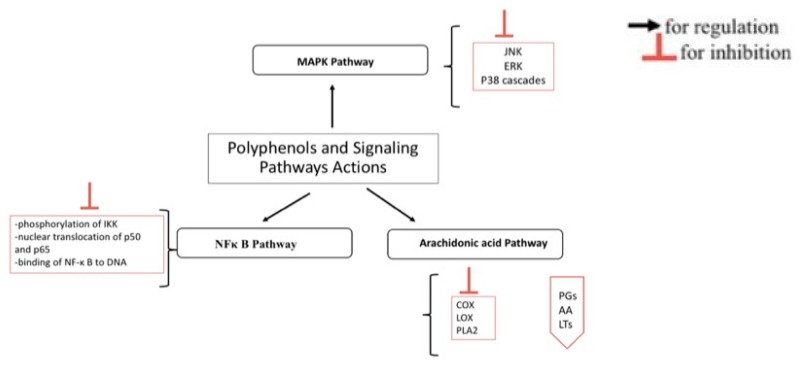
Potential points of action of polyphenols within inflammatory cascade. NF-κ B: nuclear factor kappa-light-chain-enhancer of activated B cells; IKK: IkB-kinase; ERK: extracellular signal-related kinases; JNK: c-Jun amino-terminal kinases; p38 (or p38-MAPK): p38-mitogen-activated protein kinase; COX: cyclooxygenase; LOX: lipoxygenase; AA: arachidonic acid; PLA2: phospholipase A2; PGs: prostaglandins; LTs: leukotriens. For references see the text.

NFκ B is located in the cytoplasm, it exists as an inactive non-DNA-binding form. Iκ B proteins (Iκ Bs), are inhibitors proteins that are associated with NFκ B resulting in its inactivation. Iκ Bs include Iκ Bα, Iκ Bβ, Iκ Bγ, Iκ Bε, Bcl-3, precursors p100 and p105 [72]. Under stimulatory conditions, Iκ B kinase (IKK) phosphorylate IκB proteins leading to successive ubiquitination, consequent degradation of the inhibitory proteins and release of NFκ B dimer. This later can translocate into the nucleus and prompts the expression of particular genes [72]. Different mechanisms regulate NFκ B activity as per the accumulation and degradation of Iκ B, the phosphorylation of NFκ B, the hyper-phosphorylation of IKK, and the processing of NFκ B precursors [73,74,75]. Thus, the inhibition of NFκB can be of a great benefit in controlling inflammatory conditions [76]. Several polyphenols modulate NFκ B activation and reduce inflammation [77,78]. Quercetin blocks the nuclear translocation of p50 and p65 subunits of NFκ B and represses the expression of pro-inflammatory associated genes, NOS and COX-2 in RAW264.7 macrophages [79]. It inhibits the phosphorylation of Iκ Bα protein both in vitro (using macrophages) and in vivo (using dextran sulfate sodium (DSS) rat colitis model) leading to inactivation of the NFκ B pathway [80]. In human mast cells, quercetin prevents the degradation of Iκ Bα, as well as the nuclear translocation of p65 resulting in reduction of TNFα, IL-1β, IL-6 and IL-8 [63]. It can modulate chromatin remodeling, for example it blocks the recruitment of a histone acetyl transferase called CBP/p300 to the promoters of interferon-inducible protein 10 (IP-10) and macrophage inflammatory protein-2 (MIP-2) genes in primary murine small intestinal epithelial cell. As a result, it inhibits the expression of these pro-inflammatory cytokines [81]. Quercetin can block the activation of IKK, NFκ B, and it reduces the ability of NFκ B to bind DNA in microglia treated by LPS and IFN-γ in mouse BV-2 microglia [82]. Luteolin, too, blocks NFκ B activation and inhibits pro-inflammatory genes expression and the cytokines production in murine macrophages RAW 264.7 and mouse alveolar macrophages; it also inhibits IKKs in LPS-induced epithelial and dendritic cells [83]. In addition, in co-cultured intestinal epithelial Caco-2 and macrophage RAW 264.7 cells, luteolin represses NF-ĸ B activation and TNF-α secretion [84]. Likewise, Genistein represses LPS-induced activation of NF-ĸ B in monocytes and reduces the inflammation by inhibiting NF-ĸ B activation upon adenosine monophosphate activated protein kinase stimulation in LPS-stimulated macrophages RAW 264.7 [83,85]. Galangin, as well, stops the degradation of Iĸ Bα and the translocation of p65 NF-ĸ B, repressing the expression of TNF-α, IL-6, IL-1β, and IL-8 in mast cell [86]. EGCG counteracts the activation of IKK and the degradation of Iκ Bα and inhibits NFκ B in culture respiratory epithelial cells and in vivo in male Wistar rats [87,88]. Furthermore, EGCG blocks DNA binding of NFκ B which reduces the expression of IL-12p40 and iNOS in murine peritoneal macrophages [89,90]. Catechin and epichatechin reduce NFκ B activity in PMA-induced Jurkat T cells. Flavonoids can modulate NFκ B activation cascade at early phases by affecting IKK activation and regulation of oxidant levels or at late phases by affecting binding of NF-κ B to DNA in jurkat Tcells [91]. Hydroxytyrosol, and resveratrol inhibit NFκ B activation, and the expression of VCAM-1 in LPS-stimulated human umbilical vein endothelial cells [92]. In summary, polyphenols can modulate NFκ B activation cascade at different steps such as by affecting IKK activation and regulating of the oxidant levels or by affecting binding of NF-κ B to DNA leading to an important anti-inflammatory effect responsible for their potential value in treating chronic inflammatory conditions (Figure 1).

### 4.2. MAPK Signaling Pathway

The mitogen-activated protein kinases (MAPK) are a highly conserved family of serine/threonine protein kinases. They play a key role in a range of fundamental cellular processes like cell growth, proliferation, death and differentiation. They regulate gene transcription and transcription factor activities involved in inflammation. Extracellular signal-related kinases, like (extracellular signal-related kinases (ERK))-1/2, c-Jun amino-terminal kinases (JNK1/2/3), p38-MAP kinase (α, β, δ, and γ), and ERK5 are different groups of MAPKs expressed in mammals. These are later activated by MAP kinase kinases (MAPKK) which might be triggered by some MAPKK kinases (MAPKKK) [93]. MAPK, in its turn, cross-talks with other pathways such as NFκB, thus the complexity of the MAPK signaling pathway and its interactions. Stress and mitogens activate MAPK signaling: For example, ERK1/2 route is triggered by mitogens and growth factors while JNK and p38 cascade are stimulated by stress [94,95,96,97]. Preclinical data propose an anti-inflammatory role of JNK and p38 cascades inhibitors [98,99].

Polyphenols’ activity is specific, it depends on the cell types as well as the structure of the polyphenol itself [100]. Polyphenols can block TNF α release by modulating MAPK pathway at different levels of the signaling pathway. Luteolin reduces TNFα liberation by LPS-activated mouse macrophages, it blocks ERK1/2 and p38phosphorylation [100]. In epithelial cells, luteolin, as well as other polyphenols such as chrysin and kaempferol block TNFα triggered ICAM-1 expression by inhibiting ERK, JNK and P38 [100,101]. Quercetin blocks the phosphorylation of ERK, JNK in THP-1 activated human monocytes, while in murine macrophages RAW 246.7 triggered by LPS it blocks the phosphorylation and the activation of JNK/SAPK (stress activated protein kinases), ERK1/2, and p38 leading to a reduction in the transcription and expression of TNF-α expression [102]. EGCG reduces inflammation in various cell types by exerting an anti-MAPK activity. It reduces IL-12 expression in LPS-activated murine macrophages by prohibiting p38 MAPK phosphorylation [89,103]. In addition, EGCG is found to play a protective role in autoimmune-induced tissue damage caused by Sjogren’s syndrome: it protects human salivary glands from TNF-α induced cytotoxicity by acting on p38 MAPK1. In vivo, in female ICR mice, EGCG inhibits phorbol ester-induced activation of NFκB and CREB (cAMP response element-binding protein—a cellular transcription factor) in mouse skin by blocking the activation of p38 MAPK [104]. Polyphenols concentration plays as well a role in their modulatory activities on signaling pathways: in human coronary artery endothelial cells, the activation of the MAPKs pathways (p38, ERK1/2, and JNK) and the repression of the plasminogen activator inhibitor by catechin and quercetin is time and dose dependent [105]. The ability of polyphenolic compounds to block MAPK pathways (Figure 1) endowed these bioactive substances with therapeutic potential to protect against inflammation.

### 4.3. Arachidonic Acid Signaling Pathway

Arachidonic acid (AA) is liberated by membrane phospholipids upon phospholipase A2 (PLA2) cleavage. Cyclooxygenase (COX) or lipoxygenase (LOX) metabolize it and produce, respectively, prostaglandins (PGs) and thromboxane A2 (TXA2) by COX, and hydroxyeicosatetraenoic acids and leukotrienes (LTs) by LOX [106]. The COX family involves different members (COX1, COX-2, and COX-3). COX-2 is responsible of the production of important quantity of prostaglandins, its expression is triggered by lipopolysaccharide and pro-inflammatory cytokines [107]. The ability of polyphenols to reduce the release of arachidonic acid, prostaglandins, and leukotrienes is considered one of their most important anti-inflammatory mechanisms (Figure 1). Their action is mainly realized by their ability to inhibit cellular enzymes, such as PLA2, COX, and LOX [21,108,109,110,111]. Quercetin blocks COX and LOX in various cell types such as rat peritoneal leukocyte, murine leukocytes, and guinea pig epidermis [110,112,113]. Similarly, red wine reduces COX-2 expression in old male F344 rats [114]. In LPS activated murine macrophages, green tea polyphenols not only suppress NF-κB and MAPK pathways but also constrain the expression of COX-2 and the release of prostaglandin (PGE2) in RAW 264.7 macrophages [115,116]. Equally, a reduction in the release of PGE2 is observed with other polyphenols, such as kaempferol in culture of LPS-stimulated human whole blood cells [117]. Extra virgin olive oil rich with more than 30 phenolic compounds inhibit 5-LOX in human activated leukocytes reducing leukotriene B4 and suppresses eicosanoids production by animal and human cells in vitro [118,119]. Finally, certain polyphenols show structural and functional similarities with specific anti-inflammatory drugs. A phenolic compound—oleocanthal—demonstrates a natural anti-inflammatory property and exhibits structural similarities to the ibuprofen (a well-known anti-inflammatory drug). Oleocanthal—like ibuprofen—inhibits COX-1 and COX-2 activities in a dose-dependent manner [120].

## 5. Polyphenols, Oxidative Stress, and Inflammation

**Figure 2:**
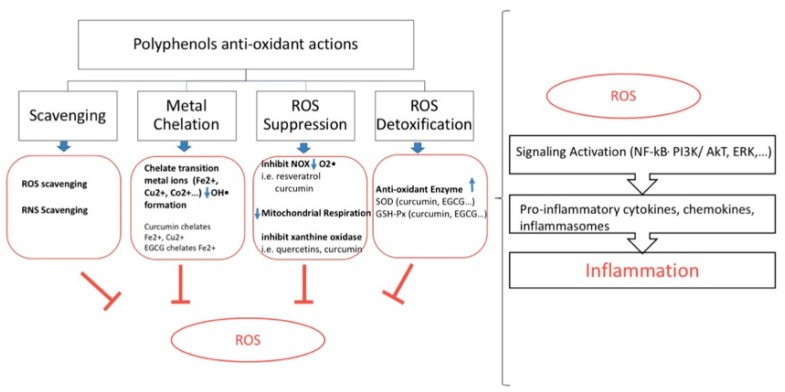
Key polyphenolic anti-oxidant actions in relation to anti-inflammation. Polyphenols scavenge radicals, chelate metal ions, inhibit ROS production and promote ROS detoxification. On the right panel ROS contribution to inflammation. ROS: reactive oxygen species; RNS: reactive nitrogen species; NOX: NADPH oxidase; SOD: superoxide dismutase; GSH-PX: glutathione peroxidase; ERK: extra-cellular signal regulated kinases; PI3K/AkT: phosphatidylinositide 3-kinases/protein kinase B; EGCG: epigallactocatechine gallate.

Higher production of reactive oxygen species (ROS) is associated with oxidative stress and protein oxidation [121]. In its turn inflammatory molecules and different inflammatory signals (i.e., peroxiredoxin2) are triggered by protein oxidations [122]. Furthermore, overproduction of ROS can prompt tissue injury that might initiates the inflammatory process [123,124,125,126,127]. Therefore, the classical antioxidant actions of polyphenols undoubtedly contribute to their anti-inflammatory roles by interrupting the ROS-inflammation cycle (Figure 2). Polyphenols are known for their antioxidant activities; they scavenge a wide-ranging selection of ROS. Polyphenols can scavenge radicals and chelate metal ions, for example quercetin chelates iron ion [128]. They also inhibit multiple enzymes responsible of ROS generation [129]. In fact, free metal ions, as well as highly reactive hydroxyl radical release, is increased by the formation of ROS. To the opposite, polyphenols are able to chelate metal ions like Fe^2+^, Cu^2+^, and free radicals which lead to a reduction of highly oxidizing free radicals [130].

Transition metal ions, like Fe^+2^, Cu^2+^, Co^2+^, Ti^3+^, or Cr^5+^, results in OH• formation from H_2_O_2_ [131,132]. Curcumin is able to chelate transition metal (Cu^2+^ and Fe^2+^) ions. Alike, EGCG and quercetin chelate Fe^2+^ (iron ion) [128]. Polyphenols like apocynin, reservatol, and curcumin can inhibit NOX (NADPH oxidase) causing a reduction in the generation of O_2_• during infections consecutively in endothelial cells in THP1-monocytes [133,134,135]. Additionally, polyphenols can attenuate the mitochondrial ATP synthesis by blocking the mitochondrial respiratory chain and ATPase. As a result, ROS production is diminished. Curcumin [136], EGCG [137], phenolic acids [138], capsaicin [139], quercetins [140], anthocyanins [140], and resveratrol analogs [141] inhibit xanthine oxidase. Thus, they reduce ROS production. Polyphenols affect the activity of cyclooxygenase, lipoxygenase, and NOS (nitric oxide synthase) as per found in RAW 264.7 macrophes [142]. These enzymes are known to metabolize arachidonic acid and their inhibition moderates the production of key mediators of inflammation (prostaglandins, leukotrienes, and NO …) [142]. Polyphenols can also restrain LPS-induced iNOS gene expression in cultured macrophages, decreasing oxidative harm [143]. Finally, they may act by upregulating endogenous antioxidant enzymes. In vivo, curcumin can stimulate antioxidant enzymes like superoxide dismutase (SOD), catalase, and glutathione (GSH) peroxidase (Px) which lead to ROS detoxification [144]. Likewise, EGCG rises SOD and GSH-Px activities with augmented amount of cellular glutathione [145]. In conclusion, polyphenols exert the anti-inflammatory action by different mechanisms: Radical scavenging, metal chelating, NOX inhibition, tempering the mitochondrial respiratory chain, inhibition of certain enzymes involved in ROS production, like xanthine oxidase and upregulation of endogenous antioxidant enzymes.

## 6. Polyphenols, Chronic Diseases and Cancer

Referring to the previously cited roles of polyphenols in maintaining tissue homeostasis by targeting different signaling pathways and referring to their antioxidant, anti-inflammation, and protection against pro-inflammation properties; polyphenols play a beneficial role in the prevention and the process of chronic diseases related to inflammation.

Various polyphenolic compounds show protective actions in diabetes, obesity, neurodegeneration, cancers, and cardiovascular diseases, among other conditions [30,146,147,148,149,150,151,152,153,154].

### 6.1. Polyphenols and Insulin Resistance

Polyphenols reduce insulin resistance. They promote glycolysis by activation of AMPK (AMP- activated protein kinase) or inhibition of mTORC1 and PI3K/AkT in vivo (in rats), ex vivo (in rats’ muscles strips) and in vitro (in C2C12 myoblasts and HELA cells) [148,149,155,156]. Additionally, AMPK activation by polyphenols increases glucose uptake by positively affecting eNOS imitating muscle contraction and in vivo activity of insulin [148,149,150]. Similarly, it is found that polyphenols lower insulin resistance by inhibiting PI3K/AkT and JNK of activation of the AMPK-SirT1-PGC1α axis (i.e., gingerol and anthocyans, and their ability to protect from diabetes and reduce insulin resistance using in vivo, ex vivo and in vitro studies [26,27,28,148,149,155]. In addition, polyphenols attenuate glucose intake from carbohydrates by inhibiting rats’ α-glucosidase [157]. Lastly, polyphenols, like falvonoids, can improve insulin secretion by reducing apoptosis of pancreatic β–cells [145].

### 6.2. Polyphenols and Inflammatory Cardiovascular Diseases (CVD)

Meta-analysis studies have reported that an intake of three cups of tea per day reduces CVD by 11% [151] while adequate intake of red wine is associated with 32% lower risk of cardiovascular disease (CVD) [158]. Soy and cocoa flavonoids contribute to the prevention of CVD as per meta-analysis of randomized controls trial [159]. Polyphenols exert their protective effects in CVD due to their anti-hypertensive potentials. Resveratrol inhibits ACE (angiotensin converting enzyme) and PDE (phosphodiesterase) and upregulates eNOS (endothelial NOS) resulting in a reduction in high blood pressure as per multiple in vivo and in vitro studies [26,27,28,155,156,160]. In addition, flavanols and flavonols exert their CVD prevention role by reducing the manifestations of age-related vascular injury. They reduce nicotinamide adenine dinucleotide phosphate (NADPH) oxidase by affecting MAPK signaling and downregulating NF-κB in aged rats [161,162,163]. At the end, the antioxidant action of polyphenols and their ability to suppress LDL oxidation leads to endothelium-protective activity [164].

Certain polyphenols like resveratrol and anthocyanins protect from CVD by multiple mechanisms; they have (1) antihypertensive properties, they inhibit eNOS, and (2) inhibit NFκB mediated expression of VCAM and ICAM expression as per previously discussed [39,165]. Polyphenols can also reduce LDL oxidation or improve LDL/HDL ratio. For example, flavanones such as hesperetin in orange juice reduce LDL/HDL ratio while quercetin inhibits LDL oxidation with elevated paraoxonase and eliminate atherogenic lesions referring to in vitro and in vivo studies (using human male subjects) [166].

### 6.3. Polyphenols and Inflammatory Neurological Diseases

Polyphenols show protective effects in neurological disease [152,153]. High flavonoid intake can reduce by 50% dementia and aging. More precisely, it lowers the incidence of Parkinson’s and delays the onset of Alzheimer’s disease as per different epidemiological studies [167,168,169,170]. EGCG has neuroprotective properties due to its antioxidant (SOD, GSHPx) activities and cellular GSH contents and ability to reduce ROS contents. Similarly, anthocyanins neuroprotective characteristics are related to the improvement of oxidative stress and reduction of Aβ deposition [38,171,172]. Other mechanisms of polyphenols protection in neurodegenerative diseases is modulation of neuronal and glial signaling pathways [173]. Polyphenols can downregulate NF-κB related with iNOS generation in glial cells [174,175,176]. Moreover, their ability to inhibit monoamine oxidase plays a positive role in cognition, depression, and learning ability in vivo in male laca mice [172].

### 6.4. Polyphenols and Inflammatory Obesity

Polyphenols exert their anti-obesity effect by activation of AMPK (5’ adenosine monophosphate-activated protein kinase) leading to a reduction of cholesterol, fatty acid synthesis, and triglyceride formation by inhibiting HMG-CoA reductase and acetyl CoA carboxylase. Furthermore, they can inhibit genes involved in adipocyte differentiation and triglyceride accumulation. They block mTORC1 and repress specific signals associated with diminished levels of PPARγ and C/EBP α/δ mRNA throughout adipogenesis (in an experimental model of sepsis) and in vitro [30,146]. They can improve energy expenditure, stop the maturation of preadipocytes into adipocytes and increase the expression of adiponectin (a hormonal protein with a role in regulating glucose levels and breaking down fatty acids). For example, capsaicin enhances the energy spending in adipose tissue. Capsaicin diminishes intracellular triglycerides and improves brown adipose tissue thermogenesis. Furthermore, in clinical studies, capsaicin is found able to increase satiety [30,177,178]. EGCG inhibits MEK/ERK and PI3K/AKT pathways leading to inactivation of preadipocytes maturation by downregulating the expression of different genes like PPARγ and C/EBPα that are associated with adipogenesis [38,41,146,179]. Certain polyphenols can increase adiponectin such as gingerol and curcumin in serum of human subjects based on randomized controlled trial [180,181].

### 6.5. Polyphenols and Cancer

Clinical and epidemiological studies have reported that polyphenols have chemo-preventive and anticancer efficacy [182,183,184]. Polyphenol compounds have the ability to inhibit the proliferation of different types of cancer such as prostate, bladder, lung, gastrointestinal, breast, and ovarian cancers [154]. For instance, quercetin, resveratrol, green tea polyphenols [185], epigallocatechin-3-gallate [186], and curcumin [187] have demonstrated efficacy as anticancer compounds. Several studies reported that polyphenols are able to prevent cancer initiation (cyto-protective), progression, recurrence, and metastasis to distant organs (cytotoxic) as per different epidemiological, in vitro, and in vivo studie [188,189,190]. However, a dichotomy exists between polyphenols’ antioxidant effects in normal cells, and their potential pro-oxidant effects in cancer cells [154,188].

Recent studies illustrated a direct correlation between ROS in intracellular signaling cascade and carcinogenesis [191]. Oxidative stress targets proteins, lipids, and DNA/RNA causing changes that increase the risks of mutagenesis. ROS/RNS (reactive nitrogen species) overproduction over a prolonged period of time damages cellular structure and functions and causes somatic mutations such as pre-neoplastic and neoplastic transformations that may lead to cell death by necrotic and apoptotic processes [192]. Polyphenols compounds contain hydroxyl groups that donate their protons to reactive oxygen species (ROS) [193]. Moreover, they reduce the activity of phase I enzymes, primarily cytochrome P450 enzymes (CYPs), such as CYP1A1 and CYP1B1 which lead to prevent the formation of reactive and carcinogenic metabolites in human bronchial epithelial cells [194]. They also can induce phase II enzymes that initiate the formation of polar metabolites which are readily excreted from the body [195]. Certain dietary polyphenols such as flavonoids reduce cellular formation of ROS which protects from the oxidation of DNA [193].

In addition to their anti-oxidant properties, pro-oxidant characteristic of polyphenols is important in treating and preventing cancer. Pro-oxidant activity can be initiated by certain conditions such as superoxide leakage [196]. The pro-oxidant activities of polyphenols in cancer cells can result in inducing apoptosis [197], cell cycle arrest [198] and inhibiting the proliferation signaling pathways (i.e., epidermal growth factor receptor/mitogen activated protein kinase, phosphatidylinositide 3-kinases/protein kinase B, as well as NF-ĸB) [199]. For example, polyphenols from apple are able to inhibit the proliferation of human bladder transitional cell carcinoma (TCC, TSGH-8301 cells), inducing G2/M cell cycle arrest, and promoting apoptosis [200]. In human papilloma virus-18-positive HeLa cervical cancer cells, green tea polyphenols can induce cell cycle arrest at the subG1 phase and apoptosis through caspases activation [201]. Flavonoids, such as quercetin, induce apoptosis in many cancer cells such as leukemic U937 cell [202], prostate cancer cells [203], hepatic cancer cells [204], among other types. A combination of quercetin with resveratrol and catechin inhibits breast cancer progression in vitro and in vivo by inducing apoptosis in carcinogenic breast cells [205]. In addition, polyphenols can reduce cancer metastasis such as quercetin [206,207].

**Figure 3:**
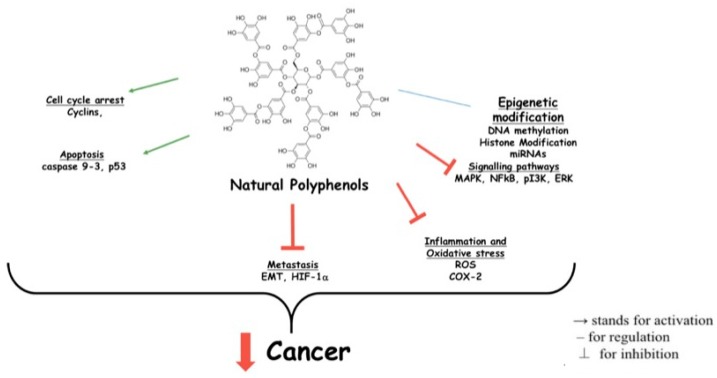
Anti-tumorigenic activities of polyphenols. MAPK: mitogen-activated protein kinase; NFκB: nuclear factor kappa-light-chain-enhancer of activated B cells; PI3K: phosphatidylinositide 3-kinase; ERK: extracellular signal-related kinases; ROS: reactive oxygen species; COX: cyclooxygenase; EMT: epithelial mesenchymal transition; HIF-1α: hypoxia-inducible factor 1-aplha.

Sufficient studies have reported that NF-κB signaling pathways are closely related to cancer metastasis. Polyphenols can disrupt the metastatic potential of cancer by inhibiting NF-κB activity [208]. Curcumin is a good example [209,210,211] of decreasing cancer metastasis in mice by suppressing NF-κB expression and down-regulating VEGF (vascular endothelial growth factor), COX-2, and MMP-9 (matrix metallopeptidase-9) expression in tissues of the breast, brain, lung, liver, and spleen [212,213]. Moreover, the strength of metastasis is associated to the epithelial-to-mesenchymal transition (EMT) [214]. There is robust evidence that polyphenols compounds can modulate EMT and its related signaling pathways [215]. For example, EGCG, a flavan-3-ol, induces apoptosis and significantly reduces colony formation and cell migration in nasopharyngeal carcinoma (NPC) and cancer stem cells (CSC) in different cell lines [216]. Luteolin and quercetin reverse the migration and invasiveness of metastatic cells by reducing the expression of mesenchymal markers and transcriptional factors on the cell membrane (i.e., twist, snail, and N-cadherin) and upregulating adhesion molecules such as E-cadherin [217]. Thus, through variable mechanisms, polyphenols broadly downregulate inflammation origination, progression, and evolution to cancers (Figure 3).

In order to emphasize on the beneficial health effects of polyphenols, different medications containing polyphenols are FDA-approved as pharmaceutical drugs. Polyphenon^®^ E, a standardized green tea polyphenol preparation, is an FDA-approved medication to treat genital warts [218]. Another significant event in the use of polyphenols as pharmaceuticals is the FDA approval of crofelemer (a medication rich in oligomeric proanthocyanidin) to manage HIV associated non-infectious diarrhea.

## 7. Conclusions

In conclusion, the vast number of published studies proved the immunomodulatory role of polyphenols in vivo and in vitro. Different underlying regulatory mechanisms are now well elucidated. These data highlight the promising role of polyphenols in prevention and therapy of diseases with underlining inflammatory conditions, including cancer, neurodegenerative diseases, obesity, type II diabetes, and cardiovascular diseases. However, the role of polyphenols in modulating multiple inflammatory cellular pathways should be further investigated. Many questions remain unanswered about the usage of polyphenols in clinical setting. The role of the microbiota in degrading these polyphenols should be further studied. The notion of bioavailability and its impact on biofunctionality should also be revisited. It is generally believed that polyphenol activity is principally located in the gut where their immunoprotective and anti-inflammatory activities are initiated and subsequently ensuring systemic anti-inflammatory effects. Since different polyphenols can have multiple intracellular targets, additional data is needed to determine the consequences of the interaction or the synergistic effects between multiple polyphenolic compounds or polyphenols and commonly used medications. Moreover, further in vivo and meta-analysis studies in humans are necessary to fully reveal the mechanisms of action of polyphenols in several physiological conditions in order to produce important insights into their prophylactic and therapeutic uses.

## Abbreviations

ROS	Reactive oxygen species
COX	Cyclooxygenase
NOX	NADPH oxidase
SOD	Superoxide dismutase
GSH	Glutathione
Px	Peroxidase
PLA2	Phospholipase A2
PGs	Prostaglandins
LTs	Leukotrienes
MAPK	Mitogen-activated protein Kinase
IKK	Inhibitor of kappa kinase
NFκB	Nuclear factor kappa-light-chain-enhancer of activated B cells
Th1	T helper 1
NK	Natural killer
DCs	Dendritic cells
ECGC	Epigallocatechin gallate
Treg	Regulatory T cells
AhR	Aryl hydrocarbon receptor
iNOS	Inducible nitric oxide synthase
LPS	Lipopolysaccharide
MCP-1	Monocyte chemoattractant protein-1
VEGF	Vascular endothelial growth Factor
IL	Interleukin
JNK	c-Jun amino-terminal kinases
CREB	cAMP response element-binding protein
AA	Arachidonic acid
AMPK	AMP-activated protein kinase
ERK	Extra-cellular signal regulated kinases
ATP	Adenosine triphosphate
CVD	Cardiovascular disease
PI3K	Phosphatidylinositide 3-kinases
Akt	Protein kinase B
LDL	Low density lipoprotein
HDL	High density lipoprotein
RNS	Reactive nitrogen species
PPARγ	Peroxisome proliferator-activated receptor gamma
CYPs	Cytochrome P450 enzymes
MMP-9	Matrix metallopeptidase-9
NPC	Nasopharyngeal carcinoma
CSCs	Cancer stem cells
EMT	Epithelial-to-mesenchymal transition
FDA	Food and drug administration
ICAM	Intercellular adhesion molecule
VCAM	Vascular cell adhesion molecule
HIV	Human immunodeficiency diarrhea
HMG-CoA	3-Hydroxy-3-methyl-glutaryl-coenzyme A
VEGF	Vascular endothelial growth factor
TLR	Toll-like receptor
NO	Nitric oxide
eNOS	Endothelial nitric oxide synthase
PDE	Phosphodiesterase
ACE	Angiotensin converting enzyme
